# Sex-specific microbiome-host interactions: from infection to chronic disease—call for papers

**DOI:** 10.1128/msystems.00156-26

**Published:** 2026-03-24

**Authors:** Süleyman Yıldırım, Wenhan Zhu, Emily Cope, Steve Lindemann, Jotham Suez, Ashley Shade

**Affiliations:** 1 Istanbul Medipol University International School of Medicine & Research Institute for Health Sciences and Technologies (SABITA) 648227 , Istanbul, Turkey; 2 Vanderbilt University Medical Center 12328, Nashville, Tennessee, USA; 3 Northern Arizona University 3356, Flagstaff, Arizona, USA; 4 Purdue University 311308 , West Lafayette, Indiana, USA; 5 Johns Hopkins Bloomberg School of Public Health 25802 , Baltimore, Maryland, USA; 6 Centre National de la Recherche Scientifique (CNRS), Institute of Ecology and the Environment, Laboratoire Ecologie Microbienne UMR5557, Universite Lyon 133614 , Lyon, France

## EDITORIAL

Biomedical research has long documented sex-biased outcomes in infection (1), immunity (2), and chronic diseases (3–6). However, most microbiome research continues to model host-microbe interactions without treating sex as a core biological variable, despite the microbiome’s central role in regulating host physiology. As a result, the averaging of sex-dependent biological processes obscures how microbiome-host interactions shape immune regulation, disease susceptibility, and therapeutic response, limiting progress toward microbiome-informed precision medicine.

Growing evidence now indicates that biological sex is not a peripheral modifier but a core determinant of microbiome-host interactions. Sex hormones shape microbial community structure and host immune tone (6–10). Together with the microbiome, they influence key host metabolic pathways and circulating metabolites (4, 11–13), while microbial enzymes and metabolites reciprocally modulate circulating hormone levels (14–16). This bidirectional sex hormone-microbiome axis provides a mechanistic framework for understanding how endocrine and microbial signals are integrated in host physiology. These interactions help explain sex-biased phenotypes observed across infections, autoimmune and cardiometabolic diseases, cancer immunotherapy, neurodegeneration, and aging. Importantly, these interactions operate across scales, from molecular and metabolic pathways to immune regulation and clinical outcomes, underscoring the need for systems-level approaches that explicitly incorporate sex as a biological variable.

Sex-specific microbiome-host interactions are particularly evident in immunity and infection. Females generally mount stronger innate and adaptive immune responses than males, a feature that can enhance pathogen clearance but also increase susceptibility to immunopathology. Experimental and clinical studies suggest that the microbiome contributes to this balance by modulating cytokine signaling, immune cell differentiation, and inflammatory set points in a sex-dependent manner (17, 18). Furthermore, sex-biased trajectories have been reported for multiple viral infections and vaccine responses, as well as for post-infectious conditions such as long COVID (2, 19–21), highlighting the relevance of sex-aware microbiome-immune interactions not only during acute disease but also in recovery and chronic sequelae. Beyond infection, chronic non-communicable diseases provide compelling examples of sex-dependent microbiome effects. For example, autoimmune diseases disproportionately affect women, whereas cardiometabolic disorders, hypertension, and impaired glucose metabolism tend to be more prevalent or severe in men. Seminal experimental studies demonstrated that gut microbiota transfer can reverse sex-biased autoimmune phenotypes through hormone-dependent mechanisms (6), providing direct causal evidence that the microbiome mediates sexual dimorphism in disease risk. Consequently, these findings illustrate how ignoring sex-dependent microbiome structure and function can lead to incomplete or misleading mechanistic models of chronic diseases.

Addressing this gap now requires confronting the conceptual and methodological constraints that continue to shape the field, particularly in how sex is incorporated into study design and analysis. Much of the existing human evidence is cross-sectional and correlative, which has been essential for identifying sex-dependent associations but remains insufficient on its own to resolve causal mechanisms. In addition, many studies are underpowered to detect sex-specific effects, and sex is frequently treated as a nuisance variable rather than as a biological variable of interest. Collectively, these limitations reflect gaps in study design and analytical practice that can be improved, particularly given recent advances in multi-omics integration, experimental manipulation, longitudinal cohort design, and computational modeling.

## CALL FOR SUBMISSIONS

*mSystems* welcomes original Research Articles, Methods and Protocols, Observations, Hypotheses, and Perspective articles and proposals for Minireviews that advance systems-level understanding of sex-specific microbiome-host interactions. We particularly encourage rigorous, hypothesis-driven studies that explicitly treat sex as a biological variable and integrate complementary experimental and computational approaches to interrogate causal mechanisms relevant to precision medicine. Submissions may span laboratory, animal, clinical, or population-based research and may address fundamental or applied questions across host-microbiome interactions, including physiological regulation, immune function, metabolic and neurobiological processes, health across the life span, and responses to environmental or therapeutic perturbations. Studies that leverage longitudinal designs, experimental perturbations, multi-omics integration, and advanced analytical frameworks to move beyond descriptive association toward mechanistic and translational insight are especially encouraged. In addition, the focus of submitted work should clearly lie in microbiome and microbiological interactions with sex as described above (e.g., the microbial component should be treated also as a biological variable and not a descriptor).

Together, the contributions to this special collection aim to position sex-aware microbiome research as a foundational pillar of microbiome-informed precision medicine. By centering sex as a core biological variable and embracing integrative, multiscale approaches, this collection seeks to catalyze rigorous and mechanistically grounded research that improves biological stratification, enhances reproducibility, and informs the development of more effective, tailored microbiome and microbiological interventions for diverse patient populations.
